# 14-Methoxy-2,16-dioxapentacyclo[7.7.5.0^1,21^.0^3,8^.0^10,15^]henicosa-3(8),10,12,14-tetraene-7,20-dione

**DOI:** 10.1107/S1600536811028972

**Published:** 2011-07-23

**Authors:** Weicheng Lu, Chaomei Lian, Yan Yang, Yulin Zhu

**Affiliations:** a School of Chemistry and Environment, South China Normal University, Guangzhou 510006, People’s Republic of China

## Abstract

The title compound, C_20_H_20_O_5_, was synthesized from the reaction between 3-methoxysalicaldehyde and 1,3–cyclo­hexa­nedione in the presence of palladium(II) chloride. The two fused xanthene rings and one of the six-membered cyclo­hexane rings adopt envelope conformations, while the other six-membered cyclo­hexane ring is in a chair conformation. The mol­ecular packing is stabilized by weak inter­molecular C—H⋯O inter­actions.

## Related literature

For applications of xanthene derivatives, see: Banerjee & Mukherjee (1981 ▶); Lambert *et al.* (1997 ▶); Hideo (1981 ▶); Poupelin *et al.* (1978 ▶); Menchen *et al.* (2003 ▶); Ravindranath & Seshadri (1973 ▶); Bigdeli *et al.* (2007 ▶). For the construction of xanthene derivatives, see: Fan *et al.* (2005 ▶); Jin *et al.* (2004 ▶, 2005 ▶); Srihari *et al.* (2008 ▶); Wang & Harvey (2002 ▶).
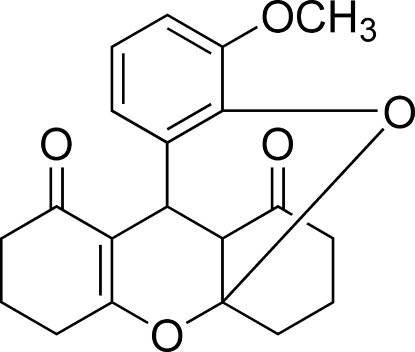


## Experimental

### 

#### Crystal data

C_20_H_20_O_5_
*M* *_r_* = 340.36
Monoclinic, 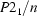

*a* = 11.0939 (15) Å
*b* = 12.5918 (17) Å
*c* = 12.2982 (16) Å
β = 104.846 (2)°
*V* = 1660.6 (4) Å^3^
*Z* = 4
Mo *K*α radiation
μ = 0.10 mm^−1^
*T* = 298 K
0.32 × 0.28 × 0.25 mm

#### Data collection

Bruker APEXII CCD diffractometer
Absorption correction: multi-scan (*SADABS*; Bruker, 2004 ▶) *T*_min_ = 0.970, *T*_max_ = 0.976
10066 measured reflections
3882 independent reflections
2361 reflections with *I* > 2σ(*I*)
*R*_int_ = 0.035

#### Refinement

*R*[*F* ^2^ > 2σ(*F* ^2^)] = 0.057
*wR*(*F* ^2^) = 0.162
*S* = 1.06
3882 reflections
231 parameters
13 restraints
H atoms treated by a mixture of independent and constrained refinement
Δρ_max_ = 0.17 e Å^−3^
Δρ_min_ = −0.19 e Å^−3^

### 

Data collection: *APEX2* (Bruker, 2004 ▶); cell refinement: *SAINT* (Bruker, 2004 ▶); data reduction: *SAINT*; program(s) used to solve structure: *SHELXS97* (Sheldrick, 2008 ▶); program(s) used to refine structure: *SHELXL97* (Sheldrick, 2008 ▶); molecular graphics: *SHELXTL* (Sheldrick, 2008 ▶); software used to prepare material for publication: *SHELXTL*.

## Supplementary Material

Crystal structure: contains datablock(s) global, I. DOI: 10.1107/S1600536811028972/rk2283sup1.cif

Structure factors: contains datablock(s) I. DOI: 10.1107/S1600536811028972/rk2283Isup2.hkl

Supplementary material file. DOI: 10.1107/S1600536811028972/rk2283Isup3.cml

Additional supplementary materials:  crystallographic information; 3D view; checkCIF report

## Figures and Tables

**Table 1:** Hydrogen-bond geometry (Å, °)

*D*—H⋯*A*	*D*—H	H⋯*A*	*D*⋯*A*	*D*—H⋯*A*
C11—H11*B*⋯O5^i^	0.97	2.57	3.538 (4)	175
C10—H10*B*⋯O3^ii^	0.97	2.59	3.466 (4)	151
C10—H10*A*⋯O1^iii^	0.97	2.40	3.367 (3)	175
C3—H3*A*⋯O3^ii^	0.97	2.52	3.389 (3)	149

Symmetry codes: (i) 
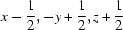
; (ii) 
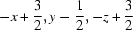
; (iii) 
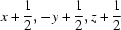
.

**Fig. 1.**
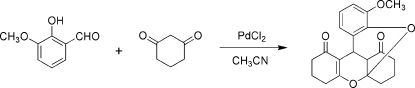
Palladium(II) chloride catalyzed synthesis of the title compound.

**Fig. 2.**
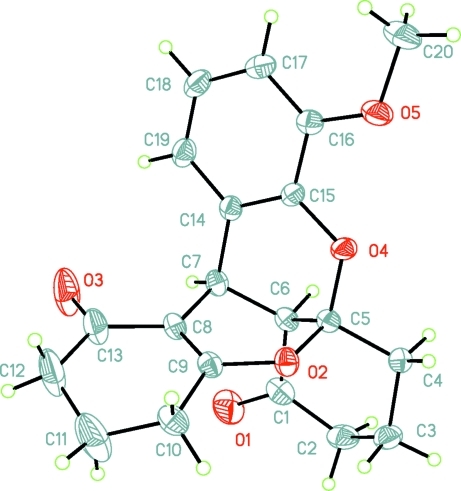
View of the title compound showing the atom–labelling scheme. Displacement ellipsoids are drawn at the 50% probability level. H atoms are presented as a small spheres of arbitrary radius.

## supplementary crystallographic information

### Comment

Xanthenes and benzoxanthenes are important biologically active heterocyclic compounds, which possess antiviral, anti–inflammatory and antibacterial activities (Banerjee & Mukherjee 1981; Lambert *et al.*, 1997; Hideo 1981; Poupelin *et al.*, 1978; Menchen *et al.*, 2003; Ravindranath & Seshadri 1973). They are also implicated in photodynamic therapy, examples including myrtucommulone–E, chromenes, rhodomyrtone (Bigdeli *et al.*, 2007). Various literature procedures are available to synthesis xanthenes (Fan *et al.*, 2005; Jin *et al.*, 2004, 2005; Srihari *et al.*, 2008; Wang & Harvey 2002). In the presence of palladium(II) chloride, the reaction between 3–methoxysalicyladehyde and 1,3–cyclohexanedione proceeded to give the title compound (Fig. 1). The molecular structure of the title compound is illustrated in Fig. 2. There are no unusual bond lengths and angles in the compound. The title molecule is built up from five fused rings *via* phenyl, xanthene, and cyclohexane. The two fused xanthene rings adopt envelope conformations, one of the six–membered cyclohexane rings is also in an envelope conformation and the other is in chair conformations. In addition, the molecules in the structure are linked *via* paired C18—H2A, O3—H10B, O1—H20B *et al.* short–contaction force.

### Experimental

A mixture of 3–methoxysalicyladehyde (0.76 g, 5 mmol), 1,3–cyclohexanedione (1.12 g, 10 mmol), and palladium(II) chloride (0.002 g) was refluxed in acetonitrile (12 ml) at 353 K for 12 h. After being cooled to room temperature, the reaction mixture was poured into water. The white precipitate was filtered off with a silica pad, washed twice with cool water, and the filtrate was then dried under vacuum to yield the product in yield of 90%. Single crystals of the title compound were obtained by slow evaporation from ethanol at room temperature to yield colourless, block–shaped crystal.

### Refinement

The H atoms were positioned geometrically and allowed to ride on their parent atoms, with C—H = 0.93–0.98Å and *U*_iso_ = 1.2 or 1.5*U*_eq_(C). Atom H7 was refined isotropically. The Δρ_max_ 0.76 (5) e^.^Å^-3^ with coordinates: 0.3847, 0.2207, 0.4643 and distance 1.09Å from C11.

### Crystal data

**Table:**

C_20_H_20_O_5_	*Z* = 4
*M**_r_* = 340.36	*F*(000) = 720
Monoclinic, *P*2_1_/*n*	*D*_x_ = 1.361 Mg m^−^^3^
Hall symbol: -P 2yn	Mo *K*α radiation, λ = 0.71073 Å
*a* = 11.0939 (15) Å	θ = 2.2–21.6°
*b* = 12.5918 (17) Å	µ = 0.10 mm^−^^1^
*c* = 12.2982 (16) Å	*T* = 298 K
β = 104.846 (2)°	Block, colourless
*V* = 1660.6 (4) Å^3^	0.32 × 0.28 × 0.25 mm

### Data collection

**Table:**

Bruker APEXII CCD diffractometer	3882 independent reflections
Radiation source: fine–focus sealed tube	2361 reflections with *I* > 2σ(*I*)
graphite	*R*_int_ = 0.035
φ–and ω–scans	θ_max_ = 27.8°, θ_min_ = 2.2°
Absorption correction: multi-scan (*SADABS*; Bruker, 2004)	*h* = −14→14
*T*_min_ = 0.970, *T*_max_ = 0.976	*k* = −16→11
10066 measured reflections	*l* = −14→16

### Refinement

**Table:**

Refinement on *F*^2^	Primary atom site location: structure-invariant direct methods
Least-squares matrix: full	Secondary atom site location: difference Fourier map
*R*[*F*^2^ > 2σ(*F*^2^)] = 0.057	Hydrogen site location: inferred from neighbouring sites
*wR*(*F*^2^) = 0.162	H atoms treated by a mixture of independent and constrained refinement
*S* = 1.06	*w* = 1/[σ^2^(*F*_o_^2^) + (0.0697*P*)^2^ + 0.3859*P*] where *P* = (*F*_o_^2^ + 2*F*_c_^2^)/3
3882 reflections	(Δ/σ)_max_ < 0.001
231 parameters	Δρ_max_ = 0.17 e Å^−^^3^
13 restraints	Δρ_min_ = −0.19 e Å^−^^3^

### Special details

**Table:**

Geometry. All s.u.'s (except the esd in the dihedral angle between two l.s. planes) are estimated using the full covariance matrix. The cell s.u.'s are taken into account individually in the estimation of s.u.'s in distances, angles and torsion angles; correlations between s.u.'s in cell parameters are only used when they are defined by crystal symmetry. An approximate (isotropic) treatment of cell s.u.'s is used for estimating s.u.'s involving l.s. planes.
Refinement. Refinement of *F*^2^ against ALL reflections. The weighted *R*–factor w*R* and goodness of fit *S* are based on *F*^2^, conventional *R*–factors *R* are based on *F*, with *F* set to zero for negative *F*^2^. The threshold expression of *F*^2^ > 2σ(*F*^2^) is used only for calculating *R*–factors(gt) etc. and is not relevant to the choice of reflections for refinement. *R*–factors based on *F*^2^ are statistically about twice as large as those based on *F*, and *R*–factors based on ALL data will be even larger.

### Fractional atomic coordinates and isotropic or equivalent isotropic displacement parameters (Å^2^)

**Table:**

	*x*	*y*	*z*	*U*_iso_*/*U*_eq_	
O2	0.96704 (13)	0.21956 (12)	0.70094 (11)	0.0371 (4)	
O4	1.10467 (13)	0.31569 (12)	0.62388 (13)	0.0395 (4)	
O5	1.33022 (14)	0.38986 (14)	0.66951 (15)	0.0513 (5)	
O1	0.66507 (16)	0.34312 (19)	0.54126 (17)	0.0729 (6)	
O3	0.7943 (3)	0.52298 (18)	0.8256 (2)	0.0935 (8)	
C6	0.88270 (19)	0.35355 (18)	0.55948 (17)	0.0342 (5)	
H6	0.9002	0.3906	0.4951	0.041*	
C5	0.98380 (18)	0.27078 (17)	0.59972 (16)	0.0327 (5)	
C8	0.87449 (19)	0.37404 (19)	0.75482 (18)	0.0359 (5)	
C14	1.0240 (2)	0.48114 (17)	0.68106 (17)	0.0338 (5)	
C4	0.9797 (2)	0.18260 (18)	0.51593 (18)	0.0391 (5)	
H4A	1.0458	0.1320	0.5462	0.047*	
H4B	0.9936	0.2118	0.4471	0.047*	
C15	1.12077 (19)	0.41893 (17)	0.66481 (16)	0.0322 (5)	
C9	0.90857 (18)	0.27250 (19)	0.77017 (16)	0.0342 (5)	
C16	1.2428 (2)	0.45815 (18)	0.68901 (18)	0.0365 (5)	
C19	1.0504 (2)	0.58235 (19)	0.72677 (19)	0.0445 (6)	
H19	0.9865	0.6244	0.7397	0.053*	
C7	0.8944 (2)	0.43368 (19)	0.65409 (18)	0.0356 (5)	
H7	0.830 (2)	0.4882 (19)	0.6303 (19)	0.046 (7)*	
C1	0.7546 (2)	0.3018 (2)	0.52006 (19)	0.0448 (6)	
C10	0.8860 (2)	0.2028 (2)	0.86036 (19)	0.0461 (6)	
H10A	0.9640	0.1906	0.9164	0.055*	
H10B	0.8545	0.1347	0.8286	0.055*	
C17	1.2662 (2)	0.55933 (19)	0.73272 (18)	0.0429 (6)	
H17	1.3468	0.5866	0.7487	0.051*	
C18	1.1706 (3)	0.6203 (2)	0.75290 (19)	0.0477 (6)	
H18	1.1877	0.6876	0.7844	0.057*	
C2	0.7457 (2)	0.2024 (2)	0.4510 (2)	0.0528 (7)	
H2A	0.7398	0.2221	0.3736	0.063*	
H2B	0.6694	0.1655	0.4525	0.063*	
C3	0.8546 (2)	0.1262 (2)	0.4899 (2)	0.0464 (6)	
H3A	0.8461	0.0893	0.5568	0.056*	
H3B	0.8520	0.0736	0.4317	0.056*	
C13	0.8222 (3)	0.4294 (2)	0.8364 (2)	0.0585 (7)	
C12	0.8086 (4)	0.3653 (3)	0.9354 (3)	0.0895 (12)	
H12A	0.8816	0.3769	0.9973	0.107*	
H12B	0.7368	0.3914	0.9587	0.107*	
C20	1.4536 (2)	0.4297 (3)	0.6828 (3)	0.0754 (9)	
H20A	1.4520	0.4869	0.6308	0.113*	
H20B	1.5063	0.3738	0.6682	0.113*	
H20C	1.4856	0.4551	0.7583	0.113*	
C11	0.7939 (4)	0.2525 (3)	0.9151 (3)	0.0989 (13)	
H11A	0.7107	0.2396	0.8679	0.119*	
H11B	0.8001	0.2171	0.9864	0.119*	

### Atomic displacement parameters (Å^2^)

**Table:**

	*U*^11^	*U*^22^	*U*^33^	*U*^12^	*U*^13^	*U*^23^
O2	0.0418 (9)	0.0381 (9)	0.0328 (8)	0.0064 (7)	0.0117 (6)	0.0029 (7)
O4	0.0262 (8)	0.0316 (9)	0.0604 (10)	−0.0017 (6)	0.0108 (7)	−0.0086 (7)
O5	0.0303 (8)	0.0486 (11)	0.0760 (12)	−0.0051 (8)	0.0157 (8)	−0.0052 (9)
O1	0.0302 (9)	0.1070 (18)	0.0784 (14)	0.0033 (10)	0.0081 (9)	−0.0151 (12)
O3	0.144 (2)	0.0657 (15)	0.0981 (17)	0.0468 (15)	0.0806 (17)	0.0133 (13)
C6	0.0312 (11)	0.0402 (13)	0.0317 (11)	0.0022 (10)	0.0087 (8)	0.0055 (10)
C5	0.0298 (11)	0.0353 (12)	0.0333 (11)	−0.0017 (9)	0.0086 (8)	0.0006 (9)
C8	0.0303 (11)	0.0427 (14)	0.0371 (12)	0.0049 (10)	0.0131 (9)	0.0012 (10)
C14	0.0410 (12)	0.0317 (12)	0.0303 (10)	0.0014 (10)	0.0124 (9)	0.0031 (9)
C4	0.0431 (13)	0.0389 (13)	0.0385 (12)	−0.0052 (11)	0.0161 (10)	−0.0053 (10)
C15	0.0371 (12)	0.0286 (12)	0.0304 (10)	−0.0025 (10)	0.0077 (8)	−0.0013 (9)
C9	0.0272 (10)	0.0448 (13)	0.0300 (10)	0.0004 (10)	0.0062 (8)	0.0009 (10)
C16	0.0359 (12)	0.0381 (13)	0.0347 (11)	−0.0023 (10)	0.0075 (9)	0.0033 (10)
C19	0.0607 (16)	0.0334 (13)	0.0428 (13)	0.0032 (12)	0.0194 (11)	−0.0011 (11)
C7	0.0326 (11)	0.0385 (13)	0.0367 (11)	0.0082 (10)	0.0105 (9)	0.0031 (10)
C1	0.0342 (12)	0.0618 (17)	0.0355 (12)	−0.0007 (12)	0.0038 (9)	0.0083 (11)
C10	0.0445 (13)	0.0553 (16)	0.0393 (12)	0.0009 (12)	0.0124 (10)	0.0111 (11)
C17	0.0480 (14)	0.0404 (14)	0.0368 (12)	−0.0133 (12)	0.0047 (10)	0.0017 (10)
C18	0.0706 (18)	0.0333 (13)	0.0397 (13)	−0.0099 (13)	0.0152 (12)	−0.0059 (11)
C2	0.0446 (14)	0.0626 (18)	0.0461 (14)	−0.0180 (13)	0.0021 (11)	−0.0013 (13)
C3	0.0536 (15)	0.0446 (15)	0.0402 (13)	−0.0148 (12)	0.0106 (11)	−0.0061 (11)
C13	0.0661 (18)	0.0611 (19)	0.0597 (17)	0.0173 (15)	0.0371 (14)	0.0048 (14)
C12	0.134 (3)	0.085 (3)	0.077 (2)	0.027 (2)	0.078 (2)	0.0141 (19)
C20	0.0366 (15)	0.081 (2)	0.112 (3)	−0.0147 (15)	0.0260 (16)	−0.014 (2)
C11	0.127 (3)	0.102 (3)	0.090 (3)	0.030 (3)	0.075 (2)	0.040 (2)

### Geometric parameters (Å, °)

**Table:**

O2—C9	1.369 (2)	C19—C18	1.375 (3)
O2—C5	1.456 (2)	C19—H19	0.9300
O4—C15	1.389 (3)	C7—H7	0.98 (2)
O4—C5	1.415 (2)	C1—C2	1.502 (4)
O5—C16	1.362 (3)	C10—C11	1.497 (4)
O5—C20	1.427 (3)	C10—H10A	0.9700
O1—C1	1.207 (3)	C10—H10B	0.9700
O3—C13	1.217 (3)	C17—C18	1.383 (3)
C6—C5	1.518 (3)	C17—H17	0.9300
C6—C7	1.520 (3)	C18—H18	0.9300
C6—C1	1.525 (3)	C2—C3	1.520 (4)
C6—H6	0.9800	C2—H2A	0.9700
C5—C4	1.508 (3)	C2—H2B	0.9700
C8—C9	1.333 (3)	C3—H3A	0.9700
C8—C13	1.459 (3)	C3—H3B	0.9700
C8—C7	1.512 (3)	C13—C12	1.501 (4)
C14—C15	1.385 (3)	C12—C11	1.445 (5)
C14—C19	1.393 (3)	C12—H12A	0.9700
C14—C7	1.513 (3)	C12—H12B	0.9700
C4—C3	1.519 (3)	C20—H20A	0.9600
C4—H4A	0.9700	C20—H20B	0.9600
C4—H4B	0.9700	C20—H20C	0.9600
C15—C16	1.400 (3)	C11—H11A	0.9700
C9—C10	1.485 (3)	C11—H11B	0.9700
C16—C17	1.381 (3)		
			
C9—O2—C5	120.06 (17)	C2—C1—C6	117.2 (2)
C15—O4—C5	118.50 (16)	C9—C10—C11	110.7 (2)
C16—O5—C20	117.6 (2)	C9—C10—H10A	109.5
C5—C6—C7	107.17 (17)	C11—C10—H10A	109.5
C5—C6—C1	111.14 (19)	C9—C10—H10B	109.5
C7—C6—C1	114.61 (18)	C11—C10—H10B	109.5
C5—C6—H6	107.9	H10A—C10—H10B	108.1
C7—C6—H6	107.9	C16—C17—C18	120.4 (2)
C1—C6—H6	107.9	C16—C17—H17	119.8
O4—C5—O2	108.62 (15)	C18—C17—H17	119.8
O4—C5—C4	107.38 (16)	C19—C18—C17	120.5 (2)
O2—C5—C4	105.67 (17)	C19—C18—H18	119.7
O4—C5—C6	112.04 (17)	C17—C18—H18	119.7
O2—C5—C6	109.72 (16)	C1—C2—C3	114.61 (19)
C4—C5—C6	113.11 (18)	C1—C2—H2A	108.6
C9—C8—C13	120.6 (2)	C3—C2—H2A	108.6
C9—C8—C7	119.78 (19)	C1—C2—H2B	108.6
C13—C8—C7	119.5 (2)	C3—C2—H2B	108.6
C15—C14—C19	119.0 (2)	H2A—C2—H2B	107.6
C15—C14—C7	118.26 (19)	C4—C3—C2	112.4 (2)
C19—C14—C7	122.7 (2)	C4—C3—H3A	109.1
C5—C4—C3	110.74 (18)	C2—C3—H3A	109.1
C5—C4—H4A	109.5	C4—C3—H3B	109.1
C3—C4—H4A	109.5	C2—C3—H3B	109.1
C5—C4—H4B	109.5	H3A—C3—H3B	107.9
C3—C4—H4B	109.5	O3—C13—C8	121.5 (2)
H4A—C4—H4B	108.1	O3—C13—C12	122.3 (2)
C14—C15—O4	123.32 (19)	C8—C13—C12	116.2 (3)
C14—C15—C16	120.9 (2)	C11—C12—C13	114.8 (3)
O4—C15—C16	115.78 (19)	C11—C12—H12A	108.6
C8—C9—O2	122.79 (19)	C13—C12—H12A	108.6
C8—C9—C10	125.2 (2)	C11—C12—H12B	108.6
O2—C9—C10	112.0 (2)	C13—C12—H12B	108.6
O5—C16—C17	125.4 (2)	H12A—C12—H12B	107.6
O5—C16—C15	115.7 (2)	O5—C20—H20A	109.5
C17—C16—C15	118.9 (2)	O5—C20—H20B	109.5
C18—C19—C14	120.2 (2)	H20A—C20—H20B	109.5
C18—C19—H19	119.9	O5—C20—H20C	109.5
C14—C19—H19	119.9	H20A—C20—H20C	109.5
C8—C7—C14	110.26 (17)	H20B—C20—H20C	109.5
C8—C7—C6	107.20 (19)	C12—C11—C10	115.4 (3)
C14—C7—C6	108.63 (17)	C12—C11—H11A	108.4
C8—C7—H7	110.3 (13)	C10—C11—H11A	108.4
C14—C7—H7	111.4 (14)	C12—C11—H11B	108.4
C6—C7—H7	108.9 (13)	C10—C11—H11B	108.4
O1—C1—C2	122.9 (2)	H11A—C11—H11B	107.5
O1—C1—C6	119.9 (2)		
			
C15—O4—C5—O2	−89.0 (2)	C9—C8—C7—C14	−86.9 (3)
C15—O4—C5—C4	157.18 (18)	C13—C8—C7—C14	90.9 (3)
C15—O4—C5—C6	32.4 (2)	C9—C8—C7—C6	31.1 (3)
C9—O2—C5—O4	96.3 (2)	C13—C8—C7—C6	−151.0 (2)
C9—O2—C5—C4	−148.79 (18)	C15—C14—C7—C8	86.9 (2)
C9—O2—C5—C6	−26.5 (2)	C19—C14—C7—C8	−89.8 (2)
C7—C6—C5—O4	−61.2 (2)	C15—C14—C7—C6	−30.4 (3)
C1—C6—C5—O4	172.89 (16)	C19—C14—C7—C6	153.0 (2)
C7—C6—C5—O2	59.6 (2)	C5—C6—C7—C8	−60.8 (2)
C1—C6—C5—O2	−66.4 (2)	C1—C6—C7—C8	63.0 (2)
C7—C6—C5—C4	177.27 (17)	C5—C6—C7—C14	58.3 (2)
C1—C6—C5—C4	51.3 (2)	C1—C6—C7—C14	−177.87 (18)
O4—C5—C4—C3	177.08 (18)	C5—C6—C1—O1	140.7 (2)
O2—C5—C4—C3	61.3 (2)	C7—C6—C1—O1	19.0 (3)
C6—C5—C4—C3	−58.8 (2)	C5—C6—C1—C2	−42.0 (3)
C19—C14—C15—O4	177.46 (19)	C7—C6—C1—C2	−163.7 (2)
C7—C14—C15—O4	0.7 (3)	C8—C9—C10—C11	13.6 (4)
C19—C14—C15—C16	−2.8 (3)	O2—C9—C10—C11	−165.0 (3)
C7—C14—C15—C16	−179.54 (18)	O5—C16—C17—C18	−177.6 (2)
C5—O4—C15—C14	−1.4 (3)	C15—C16—C17—C18	0.5 (3)
C5—O4—C15—C16	178.88 (17)	C14—C19—C18—C17	0.9 (3)
C13—C8—C9—O2	−174.7 (2)	C16—C17—C18—C19	−1.9 (3)
C7—C8—C9—O2	3.1 (3)	O1—C1—C2—C3	−142.9 (2)
C13—C8—C9—C10	6.9 (4)	C6—C1—C2—C3	39.8 (3)
C7—C8—C9—C10	−175.3 (2)	C5—C4—C3—C2	54.5 (3)
C5—O2—C9—C8	−5.9 (3)	C1—C2—C3—C4	−45.3 (3)
C5—O2—C9—C10	172.67 (17)	C9—C8—C13—O3	177.6 (3)
C20—O5—C16—C17	−7.7 (3)	C7—C8—C13—O3	−0.2 (4)
C20—O5—C16—C15	174.2 (2)	C9—C8—C13—C12	−0.6 (4)
C14—C15—C16—O5	−179.88 (18)	C7—C8—C13—C12	−178.4 (3)
O4—C15—C16—O5	−0.1 (3)	O3—C13—C12—C11	154.9 (4)
C14—C15—C16—C17	1.9 (3)	C8—C13—C12—C11	−26.9 (5)
O4—C15—C16—C17	−178.37 (19)	C13—C12—C11—C10	48.7 (5)
C15—C14—C19—C18	1.4 (3)	C9—C10—C11—C12	−41.1 (4)
C7—C14—C19—C18	178.0 (2)		

### Hydrogen-bond geometry (Å, °)

**Table:**

*D*—H···*A*	*D*—H	H···*A*	*D*···*A*	*D*—H···*A*
C11—H11B···O5^i^	0.97	2.57	3.538 (4)	175.
C10—H10B···O3^ii^	0.97	2.59	3.466 (4)	151.
C10—H10A···O1^iii^	0.97	2.40	3.367 (3)	175.
C3—H3A···O3^ii^	0.97	2.52	3.389 (3)	149.

Symmetry codes: (i) *x*−1/2, −*y*+1/2, *z*+1/2; (ii) −*x*+3/2, *y*−1/2, −*z*+3/2; (iii) *x*+1/2, −*y*+1/2, *z*+1/2.
